# Drug screening with zebrafish visual behavior identifies carvedilol as a potential treatment for an autosomal dominant form of retinitis pigmentosa

**DOI:** 10.1038/s41598-021-89482-z

**Published:** 2021-06-01

**Authors:** Logan Ganzen, Mee Jung Ko, Mengrui Zhang, Rui Xie, Yongkai Chen, Liyun Zhang, Rebecca James, Jeff Mumm, Richard M. van Rijn, Wenxuan Zhong, Chi Pui Pang, Mingzhi Zhang, Motokazu Tsujikawa, Yuk Fai Leung

**Affiliations:** 1 Department of Biological Sciences, Purdue University, West Lafayette, IN 47907 USA; 2 Purdue University Life Sciences Program, Purdue University, West Lafayette, IN 47907 USA; 3 Department of Medicinal Chemistry and Molecular Pharmacology, Purdue University, West Lafayette, IN 47907 USA; 4 Department of Statistics, University of Georgia, Athens, GA 30602 USA; 5 Department of Statistics and Data Science, University of Central Florida, Orlando, FL 32816 USA; 6 Wilmer Eye Institute, John Hopkins School of Medicine, Baltimore, MD 21205 USA; 7 Department of Ophthalmology and Visual Sciences, Chinese University of Hong Kong, Hong Kong, China; 8 Joint Shantou International Eye Center, Shantou University and the Chinese University of Hong Kong, Shantou, China; 9 Department of Ophthalmology, Osaka University Graduate School of Medicine, Osaka, Japan; 10 Department of Clinical Laboratory and Biomedical Sciences, Osaka University Graduate School of Medicine, Osaka, Japan; 11 Department of Biochemistry and Molecular Biology, Indiana University School of Medicine Lafayette, 625 Harrison Street, West Lafayette, IN 47907 USA; 12 Purdue Institute for Integrative Neuroscience, Purdue University, 610 Purdue Mall, West Lafayette, IN 47907 USA; 13 Purdue Institute for Drug Discovery, Purdue University, 610 Purdue Mall, West Lafayette, IN 47907 USA

**Keywords:** Neuroscience, Visual system, Retina, Neurodegeneration, Drug discovery, Drug screening, Phenotypic screening

## Abstract

Retinitis Pigmentosa (RP) is a mostly incurable inherited retinal degeneration affecting approximately 1 in 4000 individuals globally. The goal of this work was to identify drugs that can help patients suffering from the disease. To accomplish this, we screened drugs on a zebrafish autosomal dominant RP model. This model expresses a truncated human *rhodopsin* transgene (Q344X) causing significant rod degeneration by 7 days post-fertilization (dpf). Consequently, the larvae displayed a deficit in visual motor response (VMR) under scotopic condition. The diminished VMR was leveraged to screen an ENZO SCREEN-WELL REDOX library since oxidative stress is postulated to play a role in RP progression. Our screening identified a beta-blocker, carvedilol, that ameliorated the deficient VMR of the RP larvae and increased their rod number. Carvedilol may directly on rods as it affected the adrenergic pathway in the photoreceptor-like human Y79 cell line. Since carvedilol is an FDA-approved drug, our findings suggest that carvedilol can potentially be repurposed to treat autosomal dominant RP patients.

## Introduction

Retinitis Pigmentosa (RP) is a mostly incurable retinal-degenerative disease affecting approximately 1 in 4000 individuals globally^1–3^. Non-syndromic RP is caused by multiple mutations found in at least 65 causative genes with different modes of inheritance, while 271 causative genes have been identified in all RP subtypes. (RetNet database: https://sph.uth.edu/RetNet/)^1,4–6^. Patients suffering from RP have a cost burden of over $7000 per year on average higher than healthy individuals^7^. When patients lose their vision, they suffer from increased likelihood of injury, and increased anxiety and depression which decrease their quality of life^8^. Unfortunately, there are currently no effective treatment options available for the vast majority of patients suffering from the disease. Research into technologies including gene therapy, stem-cell therapy, and retinal prosthesis is being performed, however these options are still experimental and costly^9^. The only FDA-approved method for treating any form of RP is a recently developed gene therapy called Luxturna for the treatment of Lebar’s Congenital Amaurosis (LCA). Patients with biallelic *RPE65* mutations preventing normal expression of the gene can be treated with Luxturna, which delivers functional RPE65 with an adeno-associated virus^10^. While Luxturna is very effective in restoring some vision to LCA patients, they represent only a small proportion of all RP patients. In addition, the Luxturna treatment strategy aims to replace a deficient enzyme in an autosomal recessive case of RP, however, this will not work in autosomal dominant cases of RP (adRP). This highlights an urgent need for RP therapeutics that are effective and inexpensive. To address this need, we utilized an adRP zebrafish model to perform phenotypic drug screening and identified the FDA-approved drug carvedilol as a positive hit.

The zebrafish can provide a powerful system to model RP, and they have been used to model a number of human retinal-degenerative diseases^11–15^. These models include transgenic zebrafish expressing human *rhodopsin* (*RHO*) with autosomal dominant mutations found in RP patients^16^. Up to 30% of RP cases are autosomal dominant, and of all autosomal dominant cases, and approximately 30% arise due to over 150 mutations in *RHO*^5,17,18^. These mutations include Q344X/Q344ter, a truncation mutation, which shortens RHO at the C-terminus by 5 amino acids^19^*.* Patients with this mutation suffer an early onset, severe form of autosomal-dominant RP^20–22^. Q344X RHO loses a VXPX ciliary trafficking motif on the C-terminus leading to its mislocalization to the inner segment and apoptotic cell death^21,23,24^. Despite the C-terminal truncation, Q344X RHO is a catalytically active protein that is still capable of G protein signaling. It is hypothesized that mislocalized RHO in the inner segment causes aberrant ADCY signaling which would ultimately trigger apoptosis through an increase of cAMP signaling^15,25^.

In zebrafish, a transgenic model was made to express a human Q344X *RHO* in rods under the zebrafish *rho* promoter^15^. This model exhibits significant rod degeneration as early as 5 days post-fertilization (dpf). The model also possesses a nose EGFP reporter in the transgenic cassette, which allows for an efficient mutant screening starting at 2 dpf. Previous work with the Q344X zebrafish has shown that adenylyl cyclase (ADCY) inhibition can lead to modest rod survival^15^. However, it has also been shown that the activation of mislocalized RHO is not necessary to induce cell death^15,26^. These findings indicate that Q344X can cause rod degeneration through more than one mechanism. In this study, we utilized this Q344X zebrafish model to develop an in vivo drug-screening platform for identifying drugs that may treat adRP.

The zebrafish is an ideal model for in vivo screening for drugs to treat RP^9^ due to its low cost of use, high fecundity, amenability to genetic manipulation^27^. It can also facilitate RP drug discovery because of the rapid development of its visual system^28^. In particular, zebrafish rod precursors begin to differentiate into rods as early as 36 h post-fertilization (hpf) in the ventral region of the retina by expressing *rho*^29–31^. The rod outer segments begin to form by 50 hpf, and fully formed outer segments have been found as early as 4 dpf^32–34^. These rods begin to form synapses by 62 hpf^32,35^. The earliest visually-evoked startle can be detected by 68 hpf^36^. After that, several visual behaviors gradually appear from 3 to 5 dpf, including the optokinetic response and the visual motor response (VMR)^37–40^. The VMR is a startle response triggered by a sudden light onset or offset, which results in increased locomotor behavior^9,38,40–43^. This behavior can be measured from multiple larvae simultaneously in 96-well plate format and is thus ideal for high-throughput, in vivo drug screening experiments^9,44^. The VMR has been utilized to identify oculotoxic drugs, and discover drugs that can benefit retinal degeneration^44,45^. Zebrafish have also been used to perform high-throughput drug screening based on fluorescent signals in the retina, but this approach does not provide direct functional insight^46,47^. On the contrary, utilizing the VMR as a drug-screening platform identifies compounds that improve visual function. To date, visual behavior including the VMR has not been used to screen drugs to treat RP. One reason is that due to the rods are not deemed functional until around 15 dpf^30,48,49^. However, recent works have detected rod ERG, and rod-mediated VMR and optokinetic response (OKR) in fish larvae as early as 5–6 dpf^50,51^. This indicates that the rod-driven, scotopic behavior of the larval zebrafish can potentially be utilized to screen drugs to treat RP.

In this study, we utilized a scotopic VMR assay utilizing the Q344X zebrafish model to screen for drugs that can treat RP. We found that this adRP model exhibited a diminished scotopic VMR behavior by 7 dpf. This response was driven by rods, as confirmed by specific rod ablation. Since it has been suggested that oxidative stress in the retina acts as one of the extrinsic factors to RP progression^52^, we leveraged this assay to screen a Redox library to determine if modulating oxidative stress could improve vision and increase rod survival in the Q344X zebrafish. The discovery of a drug that could alleviate oxidative stress could broadly treat RP regardless of the causative mutation, and zebrafish have proved to be an effective model for investigating the effects of oxidative stress in the eye^53,54^. Our screen uncovered carvedilol, a β-adrenergic receptor antagonist, enhanced the Q344X zebrafish VMR and increased rod number. We provided evidence that this drug acted on rods autonomously. Since carvedilol is already approved by the FDA to treat heart failure and high blood pressure, this drug can potentially be repurposed for the treatment of RP.

## Results

### VMR assay utilization for drug screening on Q344X zebrafish with scotopic illumination

**Figure 1:**
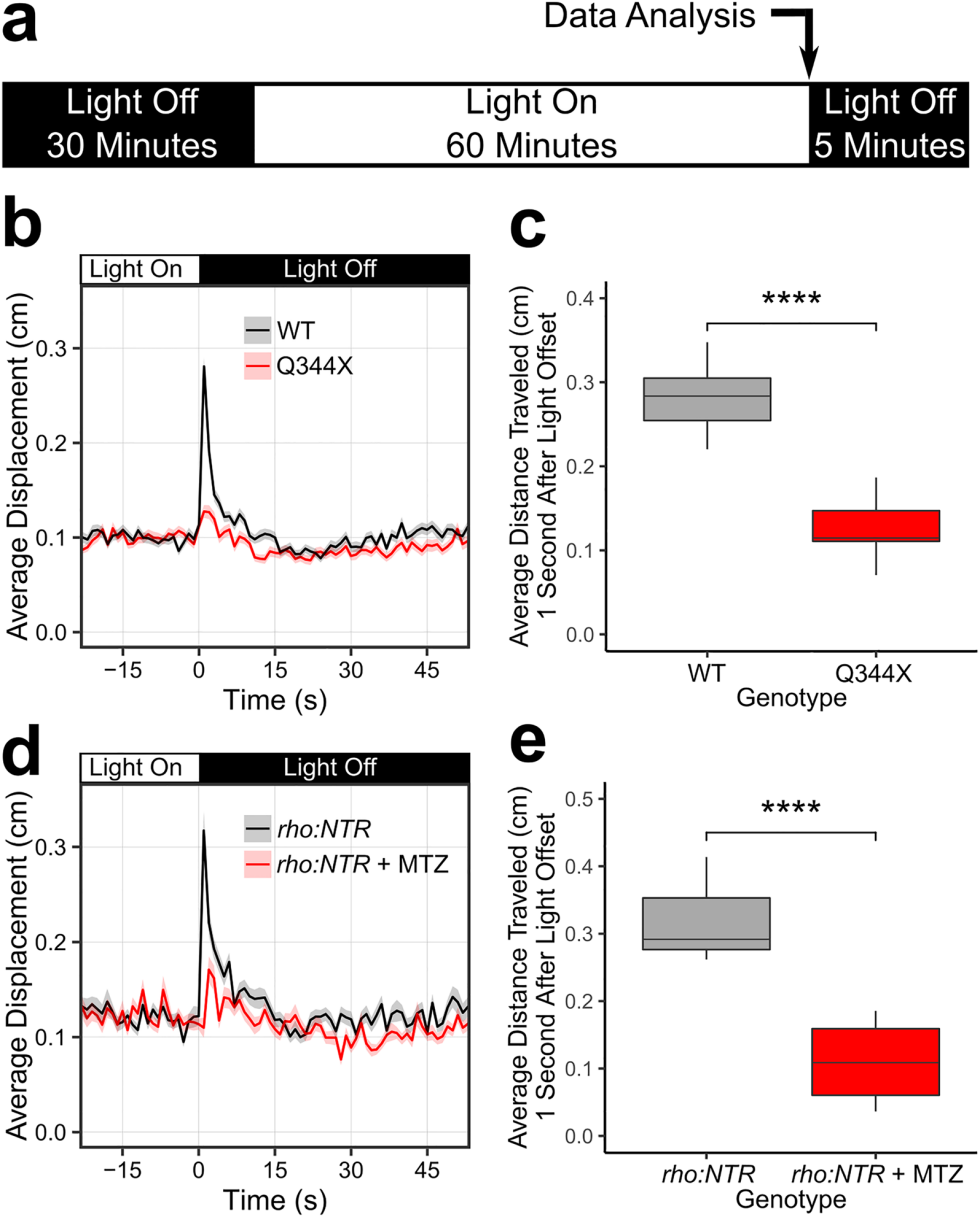
The Q344X larvae displayed a diminished scotopic light-off VMR driven by rods. (**a**) Schematic of the VMR protocol. On 7 dpf, larvae were habituated to the machine in darkness for 30 min. Then, the light stimulation was turned on and the plate was illuminated for 60 min. After that, the light was turned off. In this study, we mainly analyzed the VMR at light offset (light-off VMR) as indicated by the arrow. (**b**) The light-off VMR of wildtype (WT, black trace) and Q344X (red trace) larvae at 0.01 lx. The light was turned off at Time = 0. Each trace shows the average larval displacement of 18 biological replicates with 48 larvae per condition per replicate. The corresponding color ribbon indicates ± 1 standard error of the mean (s.e.m.). (**c**) Boxplot of the average larval displacement of WT and Q344X larvae one second after light offset. The average displacement of WT larvae (µ ± s.e.m.): 0.281 ± 0.036 cm, N = 18) was significantly larger than that of Q344X larvae (0.127 ± 0.031 cm, N = 18) (Welch’s Two Sample t-test, T = 13.2, df = 33.2, *p* value < 0.0001). To confirm this scotopic VMR was driven by rods, we chemically-ablated rods in larvae and subjected them to the same scotopic VMR assay (**d**, **e**). (**d**) The light-off VMR of larvae with nitroreductase-expressing rods treated with metronidazole (*rho:NTR* + MTZ, red trace) and without metronidazole (*rho:NTR*, black trace). Each trace shows the average displacement of 6 biological replicates with 24 larvae per condition per replicate. The corresponding color ribbon indicates ± 1 s.e.m. (**e**) Boxplot of the average displacement of *rho:NTR* and *rho:NTR* + MTZ larvae one second after light offset. The average displacement of untreated *rho:NTR* larvae (µ ± s.e.m.): 0.317 ± 0.061 cm, N = 6) was significantly larger than that of *rho:NTR* + MTZ larvae (0.110 ± 0.062 cm, N = 6) (Welch’s Two Sample t-test, T = 5.9, df = 10, *p* value < 0.0001).

We utilized the VMR assay to screen drugs with the Q344X zebrafish model using a scotopic light stimulus. This fish model was selected for our drug screening as its rods begin to degenerate at 5 dpf, and the rod degeneration becomes severe by 7 dpf^15^. This rapid rod degeneration facilitates rapid evaluation of many compounds on many individual larvae. To determine the visual consequences of rod degeneration in the Q344X zebrafish, their VMR were measured under scotopic light illumination. An appropriate scotopic intensity was identified by systematically attenuating light intensity with neutral density filters until the light was 0.01 lx (Supplementary Fig. S1). To conduct the VMR assay, Q344X transgenic larvae were identified and sorted at 2 dpf by nose fluorescence. These larvae were dark adapted overnight at 6 dpf in a 96-well plate, and their VMR assessed at 7 dpf. To conduct a VMR experiment, these larvae were acclimated to the machine in darkness for 30 min, exposed to the scotopic light of 0.01 lx for 60 min, and then exposed to darkness again (Fig. 1a). The larval displacement was recorded per second for the duration of the experiment. When exposed to a light intensity of 0.01 lx, wild-type (WT) larvae displayed a robust startle response immediately after light offset (light-off VMR), while Q344X larvae displayed a significantly diminished light-off VMR (Fig. 1b). Specifically, WT larvae traveled significantly further on average than the Q344X larvae (*µ* ± standard error of the mean (*s.e.m*): 0.281 ± 0.036 cm vs. 0.127 ± 0.031 cm) one second after light offset (Fig. 1c). Both Q344X and WT larvae did not show a response to the light onset at 0.01 lx, and both groups displayed a similar VMR at higher photopic intensities (Supplementary Fig. S2). These results indicate that the expression of Q344X *RHO* diminished the light-off VMR of Q344X larvae at 0.01 lx.

The diminished VMR of Q344X larvae was likely caused by rod degeneration. We confirmed rods were responsible for the diminished scotopic VMR of Q344X larvae by rod ablation. To this end, we utilized a zebrafish line expressing *nitroreductase* (*NTR*) specifically in rods under the control of the *rhodopsin* promotor (*rho:NTR*)^46^. This enzyme would convert a prodrug metronidazole (MTZ) into a cytotoxic substance and specifically ablate rods. In this study, the NTR-expressing larvae were treated with 2.5 mM MTZ (*rho:NTR* + MTZ) from 5 to 7 dpf, and their scotopic light-off VMR was measured at 7 dpf. Like the Q344X line, the rod-ablated larvae showed a significantly diminished light-off VMR compared with the untreated larvae (Fig. 1d). The average displacement of *rho:NTR* group (0.317 ± 0.061 cm) was significantly further than that of *rho:NTR* + MTZ group (0.110 ± 0.062 cm) (Fig. 1e). The reduction of scotopic light-off VMR by rod ablation indicates that the response was substantially driven by rods. The *rho:NTR* line displayed a strong VMR to photopic stimuli with and without MTZ treatment indicating the cone pathway is not ablated and intact (Supplementary Fig. S3). This scotopic light-off VMR was then used to screen drugs that might improve rod response with the Q344X zebrafish model.

### Drug screening revealed that carvedilol ameliorates the attenuated Q344X VMR

**Figure 2:**
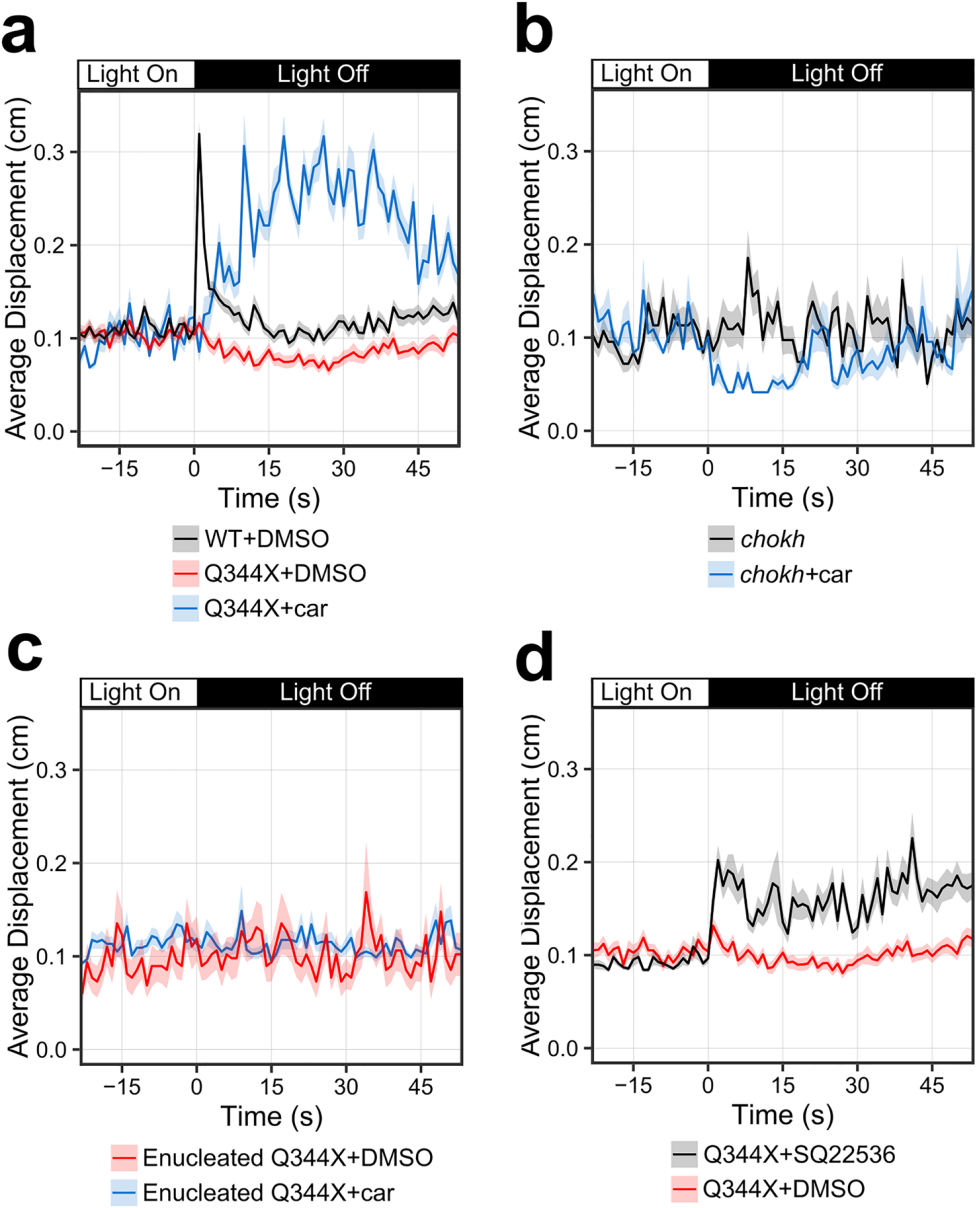
Drug screening on the Q344X zebrafish identified carvedilol as a beneficial drug. (**a**) Carvedilol treatment on Q344X larvae resulted in a sustained scotopic light-off VMR (blue trace, N = 2 replicates of 24 larvae) compared to that of both DMSO-treated WT larvae and DMSO-treated Q344X larvae (black and red trace respectively, N = 9 replicates of 48 larvae in each group). Each trace shows the average displacement of each replicate, and the color ribbons indicate µ ± s.e.m. The two carvedilol replicates were highly consistent and not different from each other (High-Dimensional Nonparametric Multivariate Test, N = 24, T_HD_ = 1.78, *p* value = 0.91). Each replicate demonstrated a significant change in behavior for the duration of 30 s after light offset above DMSO-treated Q344X larvae (Hotelling’s T-squared test, N = 24, T = 378.0 and 456.0, df = 30, *p* value < 0.0001 for each replicate). (**b**) To determine if carvedilol’s effects are elicited through the retina, eyeless *chokh* fish were treated with carvedilol (blue trace) and their VMR was compared with untreated control (black trace). Carvedilol treatment did not increase the *chokh* VMR (Hotelling’s T-squared test, N = 24 larvae, T = 37.8, df = 30, *p* value = 0.946). (**c**) Q344X larvae were enucleated to determine if extraocular expression of Q344X RHO was causing the VMR seen with carvedilol treatment. Larvae were treated with carvedilol (blue trace) or DMSO (red trace) at 5 dpf and enucleated on the morning of 6 dpf. VMR was assessed at 7 dpf. Carvedilol showed no effect on enucleated Q344X larvae. (Hotelling’s T-squared test, N = 24 larvae, T = 28.8, df = 30, *p* value = 0.948). (**d**) Q344X larvae were treated with 100 μM adenylyl cyclase (ADCY) inhibitor SQ 22,536 (black trace) at 3 dpf to determine if inhibiting ADCY would improve the VMR compared to DMSO treatment (red trace). Treatment with SQ 22,563 significantly improve the Q344X VMR over DMSO treatment (Hotelling’s T-squared test, N = 3 replicates 24 larvae, T = 118, df = 30, *p* value < 0.0001).

One of the prominent theories about RP pathogenesis is oxidative stress^52^. Since attenuating such stress might slow or prevent RP progression, we chose to screen and ENZO SCREEN-WELL REDOX library against the Q344X zebrafish model. We chose to begin drug treatment at 5 dpf to find drugs that can ameliorate the attenuated Q344X scotopic light-off VMR because rod degeneration in this model begins at this stage. 5 dpf larvae were exposed to compounds in this library dissolved in E3 media at a final concentration of 10 μM^55^, and their scotopic light-off VMR was tested at 7 dpf. The drugs of the library come dissolved in DMSO, thus all control larvae were treated with a matching concentration of 0.1% DMSO. All larvae were maintained in the same drug solution throughout the experiment. Each drug was tested twice using embryos collected on different dates. Of the 84 drugs tested, 16 were toxic to the zebrafish at 10 μM. The VMR of the remaining 68 drug-treated larval groups was normalized^56^ and then ranked based on the following selection criteria: Firstly, the two biological replicates must be consistent. The consistency was determined by a High-Dimensional Nonparametric Multivariate Test^57^ between the replicates. A small *p* value would indicate the replicates were dissimilar, whereas a high *p* value would indicate the replicates were similar. A cut off *p* value of 0.9 was chosen in this study to select those replicates that were highly similar to each other. Secondly, the drug-treated VMR must be significantly different from the DMSO-treated VMR, as determined by the Hotelling's T-squared test^40^. These criteria were applied to two timeframes: just 1 s after light offset to capture immediate response, and from 1 to 30 s after light offset to capture changes in any of the components of the VMR (Table 1). In the 1-s timeframe, 5 drug treatments gave rise to consistent larval behavior, but none of these drug treatments gave rise to a larval VMR that was significantly different from that displayed by DMSO-treated Q344X. However, in the 30-s timeframe, four drug treatments gave rise to a consistent larval behavior, and one drug treatment, carvedilol, provided both a consistent and significant change from the DMSO-treated Q344X VMR. Carvedilol-treated Q344X exhibited a sustained scotopic light-off VMR compared with DMSO-treated WT and Q344X controls (Fig. 2a). To determine if carvedilol was working through the retina, eyeless *chokh/rx3* zebrafish^58^ were treated with the drug and their VMR was assessed. The *chokh/rx3* larvae did not display a light-off VMR with or without carvedilol (Fig. 2b). Similarly, Q344X larvae were treated with carvedilol or DMSO at 5 dpf and were enucleated at 6 dpf to determine if carvedilol was exerting an effect on extraocular photoreceptors. Neither carvedilol-treated nor DMSO-treated enucleated Q344X larvae displayed a significant scotopic VMR (Fig. 2c). These results suggest that carvedilol is working at the level of the retina. Previous work with the Q344X line has shown that treatment with the ADCY inhibitor SQ 22,536 at a concentration of 100 μM improved rod survival^15^. To determine if this rod survival can translate into improved vision, the Q344X larvae were treated with 100 μM SQ 22,536 from 3 to 7 dpf at a concentration of 100 μM, and their scotopic light-off VMR was assessed at 7 dpf. The ADCY inhibitor was able to produce a significant Q344X VMR (Fig. 2d), however, this response was smaller than that produced by carvedilol treatment. Our screen therefore identified carvedilol, a drug that could functionally improve the vision of the Q344X adRP model.

**Table 1:** Summary of drug-screening results.

	1 s timeframe	30 s timeframe
Number of starting drugs in the library	84	84
Number of drugs not toxic	68	68
Number of drugs which induced consistent light-off scotopic VMR in both replicates	5	4
Number of drugs which induced consistent light-off scotopic VMR in both replicates, and significantly different from DMSO-treated controls (*p* value < 0.05)	0	1

The 84 drugs in the ENZO Redox library were each applied to the Q344X larvae at 10 μM (N = 24 larvae) in two independent replicates. Of these 84 drugs, 16 were toxic. The two replicates were then compared to each other with a High-Dimensional Nonparametric Multivariate Test to determine similarity in either 1-s or 30-s timeframe. The drugs that induced consistent light-off scotopic VMR were compared to DMSO-treated Q344X controls to determine if they caused a significant change in behavior (High-Dimensional Nonparametric Multivariate Test, *p* value < 0.05). In the 1-s timeframe, no drugs met all criteria, but in the 30-s timeframe, one drug (carvedilol) was both consistent in the two replicates and caused a significant change in light-off scotopic VMR compared to controls.

### Carvedilol treatment increased rod number in the Q344X retina

**Figure 3:**
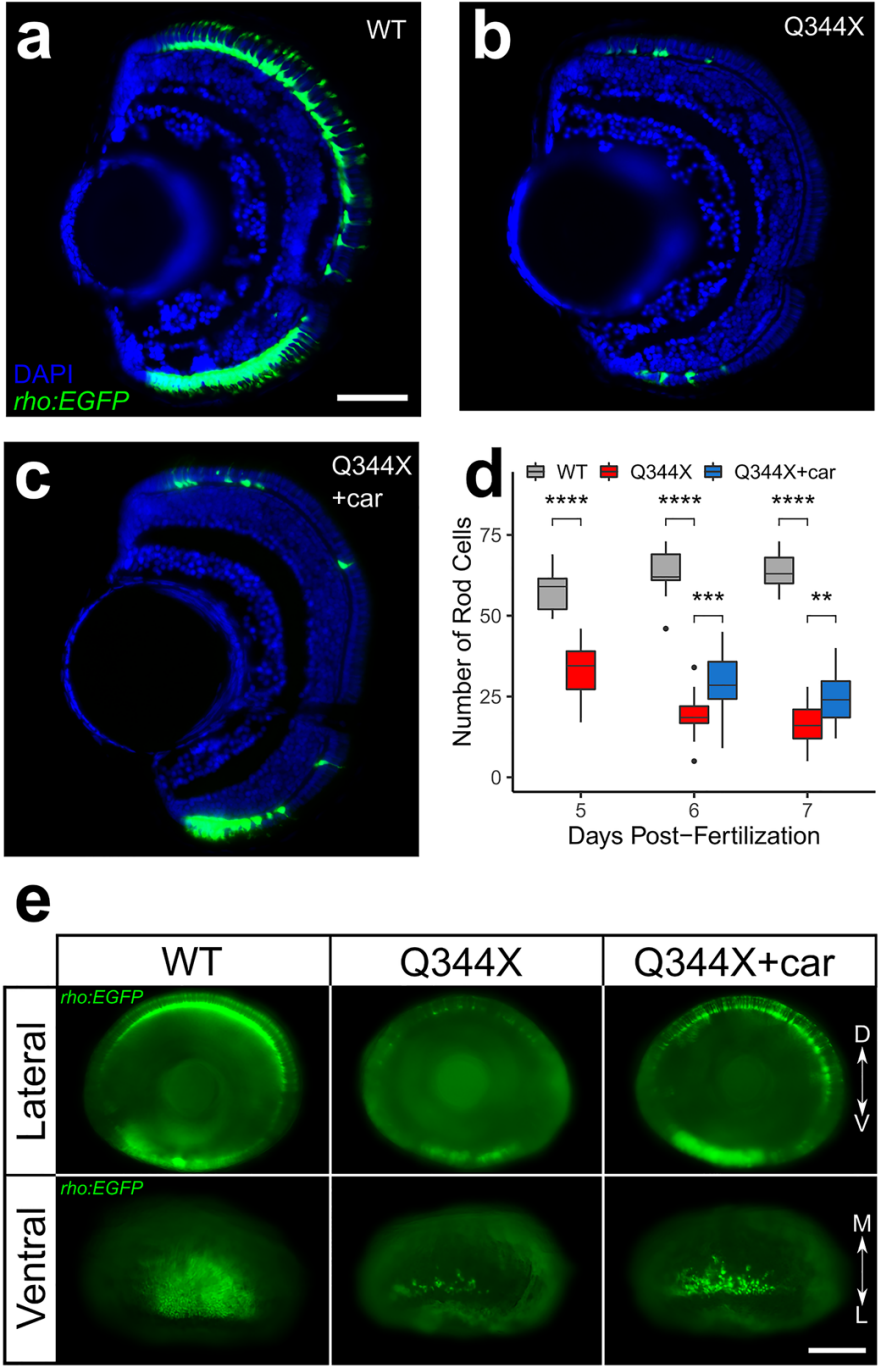
Carvedilol treatment increased rod numbers in the Q344X larvae. Representative retinal cryosection of (**a**) a wildtype larva (WT), (**b**) a DMSO-treated Q344X larva, and (**c**) a carvedilol-treated Q344X (car) larva at 7 dpf. Rods were labeled by EGFP expression driven by *rho* promoter, and the nuclei were counterstained with DAPI. Scale = 50 μm. (**d**) Quantification of rod number in WT, DMSO-treated Q344X, and carvedilol-treated Q344X retinal cryosections from 5 to 7 dpf. There was a statistically significant difference in rod number between groups at all stages determined by one-way ANOVA at 5 dpf (WT, N = 11; Q344X, N = 16; *F*(1,25) = 71.04, *p* value < 0.0001), at 6 dpf (WT, N = 9; Q344X, N = 20; Q344X + car, N = 21 ; *F*(2,44) = 96.9, *p* value < 0.0001), and at 7 dpf (WT, N = 9; Q344X, N = 17; Q344X + car, N = 11; *F*(2,41) = 167.9, *p* value < 0.0001). The effect of Q344X rod degeneration and carvedilol treatment on rod number was assessed post hoc by pairwise t-test with false discovery rate correction at 6 dpf (WT − Q344X, *p* value < 0.0001; Q344X − Q344X + car, *p* value < 0.001) and at 7 dpf (WT − Q344X, *p* value < 0.0001; Q344X − Q344X + car, *p* value < 0.001). (**e**) Representative whole-eye images of WT, Q344X, and carvedilol-treated Q344X larvae at 7 dpf. Rods were labeled by EGFP expression. Left column: WT rods were mainly found on dorsal and ventral retina (top). They were abundantly present in the ventral patch of the retina extending medially (bottom). Middle column: Q344X rods were mostly degenerated at the same stage (top). There were only a handful of rods remaining near the lateral edge of the ventral patch in the Q344X retina (bottom). Right column: carvedilol treatment increased the number of Q344X rods on both dorsal and ventral retina (top); however, gaps of missing rods were still apparently on dorsal retina. More rods were observed in the ventral patch of the carvedilol-treated retina (bottom). Statistical analysis of whole-mount data is shown in Table 2. Scale = 100 μm. *D* dorsal, *V* ventral, *M* medial, *L* lateral.

Since carvedilol enhanced the scotopic VMR of the Q344X larvae and acted through the retina, it likely exhibited benefits on the degenerating rods. The drug effect on rods was evaluated by quantification of *rho:EGFP*-positive cells on wholemount and sectioned retinae (Fig. 3). On cryosections, Q344X larvae exhibited significant rod degeneration on 5 dpf at which point they were treated with carvedilol. Carvedilol-treated Q344X larvae show increased rod number in the retina compared to DMSO-treated Q344X larvae on 6 dpf and 7 dpf (Fig. 3a–d). Next, to determine the anatomical distribution of the increased number of rods in the Q344X retina, whole-mount retinae were imaged to assess rod distribution. WT larvae had a high density of rods in the dorsal retina and ventral patch on 7 dpf while Q344X exhibited excessive rod degeneration in these areas (Fig. 3e). Carvedilol-treated Q344X showed an increased number of rods in both the dorsal retina and the ventral patch. To quantify these observations, WT, Q344X, and carvedilol-treated Q344X were binned into three classifications based on the distribution of EGFP signal: Strong, Intermediate, and Weak (Table 2). All WT larvae were classified as Strong. The carvedilol-treated Q344X larvae had significantly more Intermediate phenotypes in the lateral and ventral views compared to the DMSO-treated Q344X group. No larvae from the carvedilol or DMSO-treated Q344X groups was classified as Strong. The correlation between rod number increase and enhanced light-off VMR of Q344X larvae suggests that the increase in rod number with carvedilol treatment mediated the visual improvement.

**Table 2:** Rod analysis on whole-mount eyes.

	Strong	Intermediate	Weak
WT lateral	10	0	0
Q344X lateral	0	9	15
Q344X + car lateral	0	16	8
WT ventral	10	0	0
Q344X ventral	0	8	16
Q344X + car ventral	0	16	8

All larvae were bleached and examined from the lateral and ventral sides. The *rho:EGFP* signal were classified into 3 categories by the extent of its fluorescence. The Strong group contains the samples with high rod number/signal intensity in the dorsal retina and ventral patch; the Intermediate group contains the samples with distinct rods in the dorsal retina with noticeable gaps, and some rods in the ventral patch extending medially; and the Weak group contains the samples with sparse rods in the dorsal retina and the most lateral edge of the ventral patch. The WT image in Fig. 3e. is representative of the “Strong” group, the Q344X image in Fig. 3e. is representative of the “Weak” group, and the Q344X + car in Fig. 3e. is representative of the “Intermediate” group. Carvedilol treatment increased the number of Q344X larvae with Intermediate phenotypes and reduced the number of Weak phenotypes in both the lateral (Chi-square test, $\chi^{2}$ = 4.09, df = 1, *p* value < 0.05) and ventral views (Chi-square test, $\chi^{2}$ = 5.33, df = 1, *p* value < 0.05). No Q344X larvae was classified as Strong with or without carvedilol treatment.

**Figure 4:**
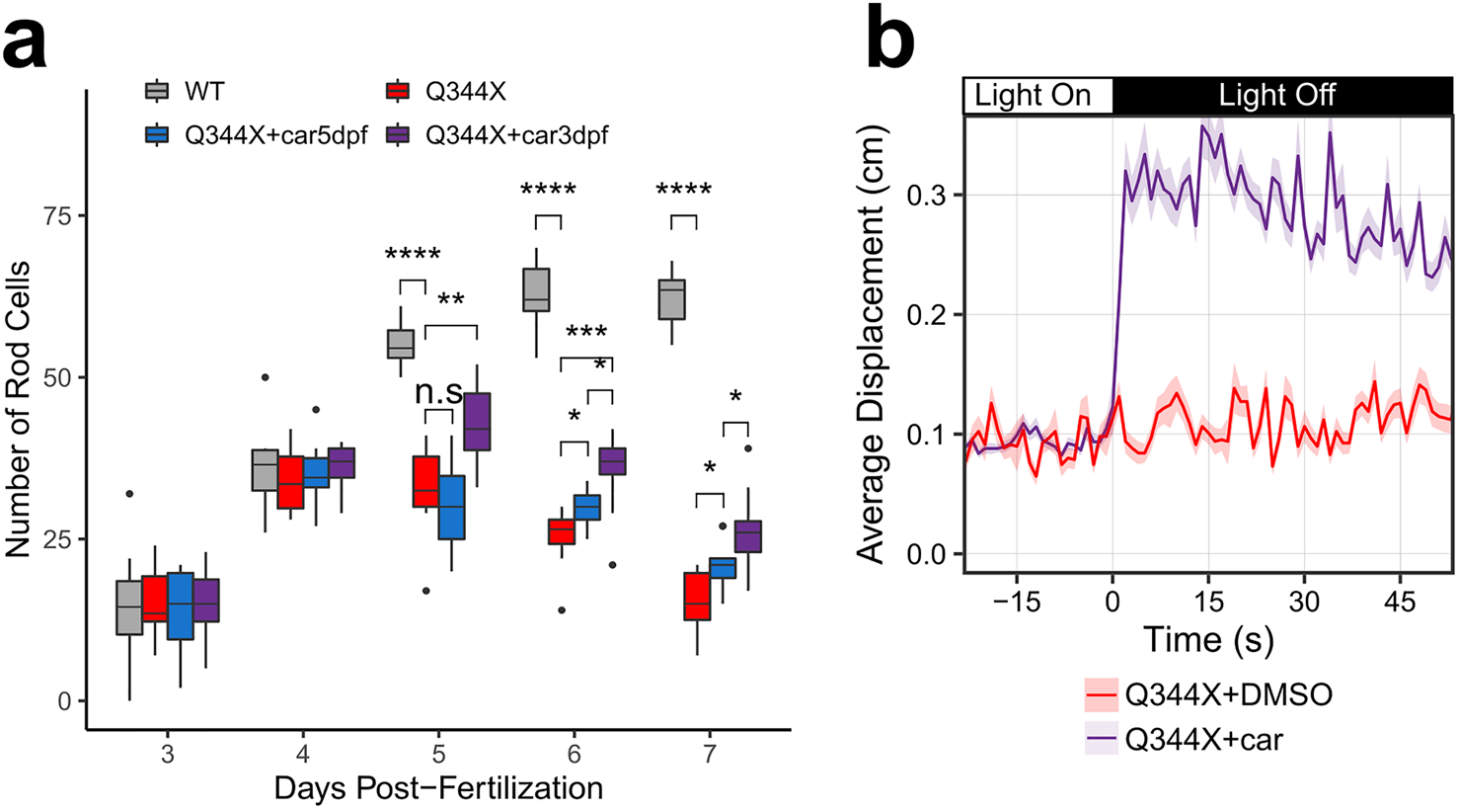
Carvedilol treatment beginning at 3 dpf increased rod numbers in the Q344X larvae greater than the treatment beginning at 5 dpf. (**a**) Quantification of rod number in WT, Q344X treated with DMSO beginning at 3 dpf, and Q344X treated with carvedilol beginning at 3 dpf or 5 dpf. Rods were quantified from their retinal cryosections beginning at 3–7 dpf. There was no statistically significant difference in rod number between groups at 3 dpf and 4 dpf determined by one-way ANOVA (3 dpf; N = 10; *F*(3,36) = 0.1, *p* value = 0.95); (4 dpf; N = 10; *F*(3,36) = 0.5, *p* value = 0.69). There was a statistically significant difference in rod number between groups at 5 dpf through 7 dpf determined by one-way ANOVA (5 dpf, N = 10; *F*(3,36) = 0.1, *p* value < 0.0001), (6 dpf, N = 10; *F*(3,36) = 0.1, *p* value < 0.0001), (7 dpf, N = 10; *F*(3,36) = 0.1, *p* value < 0.0001). The effect of Q344X rod degeneration and carvedilol treatment on rod number was assessed post hoc by pairwise t-test with false discovery rate correction at 5 dpf (WT − Q344X, *p* value < 0.0001; Q344X − Q344X + car3dpf, *p* value < 0.001; Q344X − Q344X + car5dpf, *p* value = 0.36, Q344X + car3dpf − Q344X + car5dpf, *p* value < 0.0001), at 6 dpf (WT − Q344X, *p* value < 0.0001; Q344X − Q344X + car3dpf, *p* value < 0.0001; Q344X − Q344X + car5dpf, *p* value < 0.05; Q344X + car3dpf − Q344X + car5dpf, *p* value < 0.05), and at 7 dpf (WT − Q344X, *p* value < 0.0001; Q344X − Q344X + car3dpf, *p* value < 0.0001; Q344X − Q344X + car5dpf, *p* value < 0.05; Q344X + car3dpf − Q344X + car5dpf, *p* value < 0.05). (**b**) Carvedilol treatment of Q344X larvae beginning at 3 dpf (purple trace) displayed a significant scotopic light-off VMR when compared to Q344X larvae treated with DMSO (red trace) (Hotellings T-squared test, N = 3 replicates of 24 larvae, T = 397, df = 30, *p* value < 0.0001). Each trace shows the average displacement of each replicate, and the color ribbons indicate µ ± s.e.m.

Higher doses of carvedilol were tested at 31.6 μM and 100 μM to determine if a larger treatment dose would improve rod number, but these concentrations were toxic to the zebrafish larvae. Thus, further rod number improvement was evaluated with a longer carvedilol treatment period. Q344X larvae were treated with 10 μM carvedilol beginning at 3 dpf. The drug and media were refreshed daily to maintain the health of the larvae. Larval treatment beginning at 3 dpf was compared to treatment beginning at 5 dpf to determine if earlier carvedilol treatment is more effective. There was no difference in rod number between any of the Q344X and WT groups at 3 dpf and 4 dpf indicating that Q344X rod degeneration is not significant at these stages (Fig. 4a). Q344X rod degeneration does become significant at 5 dpf, and the earlier carvedilol treatment beginning at 3 dpf significantly increased the rod number at 5 dpf (Fig. 4a). Carvedilol treatment beginning at 5 dpf with daily refreshment still improved rod number in the Q344X zebrafish at 6 dpf and 7 dpf, however carvedilol treatment beginning at 3 dpf resulted in significantly higher rod numbers than the later 5 dpf treatment (Fig. 4a.). Correlating with increased rod number, the VMR of Q344X larvae treated with carvedilol beginning at 3 dpf displayed a significantly more rapid light-off VMR (Fig. 4b) compared with the VMR of larvae treated with carvedilol treatment at 5 dpf (Hotellings T-squared test, N = 3 replicates of 24 larvae, T = 397, df = 30, *p* value < 0.0001). Carvedilol treatment beginning at 3 dpf did not have a significant effect on the photopic VMR of Q344X larvae (Supplementary Fig. S4). These results suggest that earlier carvedilol drug treatment improves the number of Q344X rods better than later treatment, and that the carvedilol effect primarily acts on the rod photoreceptors.

### Carvedilol can inhibit β-adrenergic signaling in Y79 retinoblastoma cells

**Figure 5:**
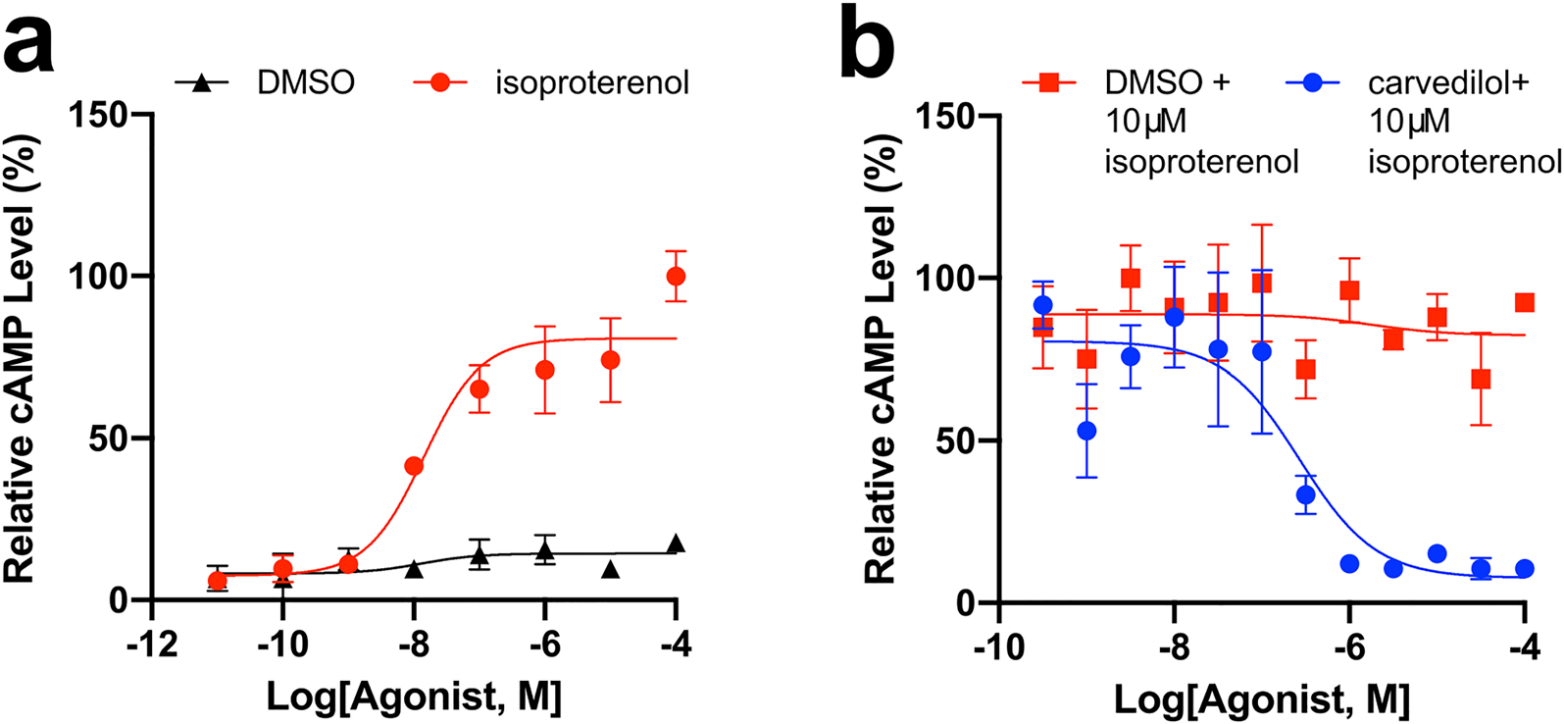
Carvedilol treatment might directly act on rods cells. To determine the extent to which carvedilol act directly on rods, we conducted a GloSensor cAMP assay with human Y79 cells. (**a**) Representative dose–response curves of GloSensor-transfected Y79 cells treated with half-log concentrations of isoproterenol (red trace; N = 4) or percentage-matched DMSO (black trace; N = 4). These plots were normalized to the maximum average luminescent level recorded per experiment. Error bars show ± 1 s.e.m. Isoproterenol was capable of increasing cAMP signaling through β-adrenergic receptor binding with an pEC50 of 7.49 ± 1.07. (**b**) Representative dose–response curves of GloSensor-transfected Y79 cells pretreated with half-log doses of carvedilol (blue trace; N = 4) or percentage-matched DMSO (red trace; N = 4). Cells were then challenged with a 10 μM isoproterenol that could induce maximal cAMP response, as shown in (**a**). Carvedilol pretreatment was capable of preventing isoproterenol-mediated cAMP increases with an pIC50 of 6.51 ± 0.67.

Carvedilol is a β-blocker that that binds to β1-adrenergic receptors, β2-adrenergic receptors, α1-adrenergic receptors and inhibits adrenergic signaling. However, its retinal target is unknown, and it may act directly on rods. To evaluate this possibility, we examined the effect of carvedilol treatment on the Y79 human retinoblastoma line which uniquely expresses rod-specific genes^59^. The Y79 cell line exists as a photoreceptor-like precursor that shows differentiation potential for the rod lineage^60^. Activin treatment of the Y79 line increases the expression of the transcription factor *Nrl* which induces progenitor differentiation into rods^60^. Previous work has leveraged this line to conduct expression studies in a photoreceptor-like cellular environment biased towards the rod lineage^61^. The level of adrenergic signaling was determined by GPCR-modulated changes in cAMP levels as measured by a cAMP-sensitive luciferase. First, the Y79 cells were transfected with the luciferase reporter, and then they were exposed to half-log dilutions of isoproterenol, a β-adrenergic receptor agonist. Isoproterenol was capable of inducing cAMP signaling in the transfected Y79 cells with a pEC50 of 7.5 ± 1.1 (Fig. 5a). The cAMP level was not increased in controls treated with matching DMSO percentage to dissolve isoproterenol. The relative cAMP level did not increase much above 10 μM isoproterenol. To determine if carvedilol treatment can inhibit this isoproterenol-mediated cAMP increase, the transfected Y79 cells were pretreated with half-log dilutions of carvedilol and then challenged with a dose of 10 μM isoproterenol that would induce saturating relative cAMP level according to Fig. 5a. Carvedilol pretreatment was able to prevent isoproterenol-mediated cAMP signaling with a pIC50 of 6.5 ± 0.7 (Fig. 5b).

## Discussion

There are no approved cures to treat the majority of RP subtypes. To address this unmet need, we have utilized the Q344X adRP zebrafish model to establish a drug screening platform that can be expanded into other subtypes of RP. We found that rod degeneration in the Q344X zebrafish resulted in a deficient scotopic light-off VMR, a locomotor response displayed during drastic light offset. This behavior was driven by rods, as it was diminished by chemical ablation of the rods. We leveraged this behavior as a functional assay to screen for beneficial drugs that could enhance the response in the Q344X model. We found that both the scotopic VMR and retinal histology of the Q344X model were improved by carvedilol. Despite showing a clear behavioral response to the scotopic light offset, the carvedilol-treated Q344X did not display a similar scotopic light-off VMR profile compared with WT. This is likely because carvedilol treatment did not restore the rod number or distribution to the WT levels. Because eyeless *chokh* larvae and enucleated Q344X larvae treated with carvedilol did not display an improved scotopic light-off VMR, it is likely that carvedilol acted at the eye level. Since carvedilol treatment of Q344X larvae shows efficacy and is approved by the FDA, studying its mechanism in the retina can potentially expedite the development of a new treatment for adRP patients.

Carvedilol has several known modes of action. It is primarily classified as a β-blocker; however, it has also been demonstrated to act as an α1-blocker, a calcium channel agonist at high concentration, and a free radical scavenger^62,63^. Carvedilol may mediate its visual benefit through some of these pathways. Traditionally, β-blockers are seen only as antagonists that prevent epinephrine from binding β-adrenergic receptors. Epinephrine is present in the mouse subretinal space and increases with light exposure^64^. Blocking epinephrine signaling can potentially lower cAMP levels in the Q344X rods by preventing endogenous ADCY signaling. Interestingly, carvedilol also acts as an atypical β-blocker which is capable of inducing biased signaling^65^. Specifically, carvedilol can promote β-arrestin signaling while acting as an inverse agonist towards G protein signaling^66^. This type of β-arrestin signaling has been shown to have anti-apoptotic effects^67–69^ that may prevent Q344X rod death. This pathway is feasible in the Q344X zebrafish as RNA-seq of adult zebrafish rods detected the expression of β_2_-adrenergic receptors^70^. Carvedilol may also exert protective effects on Q344X rods through α1 blockade since selectively blocking Gq-coupled α1 adrenergic receptors can prevent photoreceptor degeneration in a Stargardt Disease model^71,72^. In our study, it is unlikely that carvedilol is acting as a calcium channel blocker at the tested dose since it would likely stop the larval heart beating and kill the larva before its VMR could be measured^73,74^. Since our study did not identify any other beneficial compounds from this REDOX library, carvedilol probably did not exert its visual benefit on the Q344X model as a radical-scavenging antioxidant.

More evidence is available which suggests that β-blockers may be able to treat RP. A recent study has identified that another β-blocker, metipranolol, is capable improving rod survival and electroretinogram in the *rd10* mouse^75^. Another study has found that the β-blocker metoprolol can provide protection against bright light-induced retinal degeneration, and metoprolol protection can be increased by co-treatment with other GPCR agonists and antagonists^72^. Carvedilol has already been shown to have beneficial effects with treating other eye-disease models. Carvedilol can lower intraocular pressure (IOP) in the eye of rabbits^76^. Also, carvedilol has neuroprotective effects on retinal ganglion cells in an optic nerve injury mouse model^77^. However, it is unknown if β-blockers such as carvedilol can work directly on rods, so we employed the human Y79 line to determine this. We were able to demonstrate that carvedilol can inhibit isoproterenol-mediated activation of β receptors in the Y79 cells. Therefore, carvedilol likely bound to the β-adrenergic receptors and directly elicited its beneficial effects on rods. In addition, one of carvedilol’s target receptors, the β_1_-adrenergic receptor, is expressed in mouse rods^78^. These results suggest that carvedilol may be able to elicit its therapeutic effects directly on the rods, and it targets adrenergic signaling in the eye that may treat Q344X adRP.

Targeting GPCR signaling through adrenergic receptors is an attractive method for the treating Q344X adRP. While the full disease mechanism is unknown, it is believed that mislocalized activation of rhodopsin in the inner segment of Q344X rods induces ADCY activation resulting in cAMP increase and apoptosis^15,21,23,24^. This highlights ADCY as a potential drug target. Previous work with the Q344X zebrafish, as well as the Stargardt Disease mouse, has shown that inhibition of ADCY with the inhibitor SQ 22,536 improved photoreceptor survival^15,71^. SQ 22,536 treatment did improve the VMR displayed by Q344X larvae, however the overall resulting VMR was smaller than that of both carvedilol treatment conditions. Therefore, the improved Q344X VMR from carvedilol treatment may be due to other chemical properties of the drug or more efficient uptake of the drug.

We have performed the first functional drug screen for RP and have discovered carvedilol as a positive hit. Drugs identified through the presented screening method may provide a beneficial lead, but the screening parameters may not be the optimal treatment conditions for that particular drug. In the case of carvedilol, we tested a treatment period beginning at 3 dpf and determined that earlier treatment further improved rod survival. Utilizing the behavioral drug screen at 5 dpf and further investigating hits with earlier treatment is an efficient method for identifying the best hits for further translation. Investigating the VMR and rod survival at stages later than 7 dpf becomes more complicated because larval zebrafish deplete their yolk at around 9 dpf and require feeding to survive. Larval feeding and foraging introduce extra variability in the behavioral characterizations of drug effects. It may also be possible to evaluate the effect of positive drug hits by utilizing the OKR behavior of zebrafish larvae. However, it is possible for a mutant larval zebrafish to be light-sensitive and display a VMR while being incapable of displaying an OKR^38^. Emran et. al.^38^ found that the *nrc* mutant zebrafish is light sensitive while failing to produce an OKR due to disruption of ON retinal pathway. Future research will elucidate the mechanism through which these carvedilol-regulated pathways increase rod numbers, and will test carvedilol’s efficacy on the Q344X mouse model^79^ to validate the translational value of carvedilol for adRP treatment and its capability to improve vision. Positive findings in the Q344X mouse would pave the way for further screening and testing of drugs that modulate the adrenergic system to treat adRP. Our phenotypic drug screen with the Q344X zebrafish lays the foundation for drug screening with fish modeling different classes of RP mutations.

## Materials and methods

### Animals

Zebrafish of the AB background were utilized for all experiments https://zfin.org/ZDB-GENO-960809-7. The *chokh/rx3* zebrafish line (*chk*^*s399*^*)* was utilized in this study^58^. Adult and larval zebrafish were maintained and bred using standard procedure^80^. Adult fish were placed in breeding tanks the night before breeding after receiving all meals. Adult fish began spawning at 9:00am, and embryos were collected before 10:30am. Larval zebrafish were reared until 7 days post-fertilization (dpf) in E3 medium in an incubator at 28 °C. The fish incubator was kept on a 14 hr light and 10 hr dark cycle. E3 medium was changed daily, and healthy embryos were kept for experiments. All protocols were approved by the Purdue University Institutional Animal Care and Use Committee. This study was completed in compliance to the ARRIVE guidelines.

### Transgenic animals

*Tg(rho:Hsa.RH1_Q344X)* transgenic animals were generated previously^15^ and are referred to in this study as Q344X. Q344X larvae were identified on 2 dpf through the expression of EGFP under the control of 1.1 kb promoter of *olfactory marker protein* (*omp*) contained in the transgenic cassette. Their genotype was verified via PCR with the following primers: 5′-CCAGCGTGGCATTCTACATC-3′ and 5′-AACGCTTACAATTTACGCCT-3′. The rods in the Q344X line were labeled with the *Tg(-3.7rho:EGFP)* transgene^81^ and are referred to in this study as *rho:EGFP*. Zebrafish expressing *nitrodreductase* in rod photoreceptors, *Tg(-3.7rho:YFP-NfsB)*^*gmc500*^, were generated previously^46^ and referred to in this study as *rho:NTR*. To chemically ablate rods, we used the zebrafish line, *Tg(-3.7rho:YFP-NfsB)*^*gmc500*^, expressing *nitroreductase* (NTR) under the control of the rhodopsin promotor^46^ (*rho:NTR)*.

### Drug treatment

The ENZO SCREEN-WELL REDOX library was used for drug screening (ENZO Life Sciences, BML-2835-0100). Carvedilol was also ordered from ENZO Life Sciences (BML-AR112-0100) for further experiments and from MilliporeSigma (C3993-50MG) for confirming the positive effects observed in specific behavioral experiments (data not shown)*.* SQ 22,536 was purchased from Sigma (S153-5MG). All drugs tested were dissolved in DMSO. The DMSO percentage that zebrafish larvae were exposed during experiments to was 0.1% except for SQ 22,536 treatment where DMSO exposure was 1%. WT larvae were only exposed to 0.1% DMSO to control for behavioral effects in the Fig. 2a WT dataset. Thirty larvae were exposed per drug dissolved in 15 mL E3 media in a 100 × 15 mm petri dish. The treatment began on 3 dpf or 5 dpf as stated. The drug-containing media were not refreshed during experiment unless otherwise stated. The treated larvae were directly transferred into the 96-well plate with their corresponding E3 medium with drugs to ensure consistent drug dosing throughout the treatment period.

The common starting concentration of 10 μM was chosen to minimize toxic effects while maximizing the chances of finding an effective dose^55^. Drug screening with the Q344X zebrafish model begun at 5 dpf due to the onset of rod degeneration and the display of a variety of visual behaviors including the VMR and OKR^37–40^. This stage also was chosen to allow the larvae to developmentally mature to a stage as far as possible to minimize potential toxicity from drugs.

### Rod photoreceptor ablation

Treatment with the prodrug metronidazole (MTZ) specifically ablates the rod photoreceptors of the *rho:NTR* zebrafish line. Specifically, NTR-expressing larvae were treated with 2.5 mM MTZ from 5 to 7 dpf. Their VMR was compared with the untreated larvae on 7 dpf.

### Retinal histology and imaging

All larvae were fixed in 4% paraformaldehyde (PFA) overnight at 4 °C. For retinal cryosections, fixed larvae were infiltrated with 30% sucrose overnight at 4 °C prior to imbedding in Tissue Freezing Medium (GeneralData, TFM). Ten micrometers-thick cryosections were collected on Fisherbrand Superfrost Plus Microscope Slides (Thermo Fisher Scientific, 12-550-15). The sections containing the optic nerve were analyzed for anatomical reference. Rod photoreceptors were identified and quantified by utilizing the *rho:EGFP* transgene as a marker. Since the rods in the ventral retina are present in a high density, high exposure and low exposure images were captured of every cryosection to view the rods in all areas of the retina. A rod was defined as the presence of an identifiable, single soma expressing EGFP. Cryosection images presented in this study are high-exposure to visualize all of the rod signal present in the retinal slice.

### Whole-animal preparation

To visualize rod distribution in the retina, PFA-fixed larvae were bleached with 1% KOH/3% H_2_O_2_ for 40 min at room temperature to bleach the black pigment from the retinal pigment epithelium. The bleached embryos were imbedded a 3% methyl cellulose solution for observation.

### Microscope and camera

All samples were imaged with an Olympus BX51 microscope (Olympus) and a SPOT RT3 Color Slider camera (SPOT Imaging).

### Visual motor response assay

A ZebraBox system from ViewPoint Life Sciences was utilized for the Visual Motor Response (VMR) assay. Individual zebrafish larvae were placed in 96-well plate format using Whatman UNIPlate square 96-well plates (VWR, 13503-152). In order to produce a scotopic stimulus, the ZebraBox was modified to attenuate the light intensity beyond its lowest limit by fitting neutral-density filters in the light path. Seven neutral density filters (BarnDoor Film and Video Lighting, E209R), each allowing approximately 40% transmittance, were stacked between the light source and the plate holder until a final intensity of 0.01 lx was attained. In our scotopic experiments, the machine was also powered at 5% in order to prevent instability from the LED light source. The larval displacement was collected by the tracking mode which binned the activity every second.

To conduct the scotopic VMR assay, larvae were sorted and grown in 100 × 15 mm petri dishes (VWR, 25384-088) with 15 mL E3 media in a density of 30 larvae from 2 to 5 dpf. Larvae were transferred to 96-well plates with one larva per well on the morning of 6 dpf and dark adapted overnight. On 7 dpf, the dark-adapted larvae were placed in the ZebraBox and their scotopic VMR was measured. For drug screening with larvae, this procedure was the same except larvae were exposed to drugs in petri dishes on 5 dpf. In this study, the following protocol was used: 30 min in the dark followed by a 60-min scotopic light illumination at 0.01 lx, and then a light offset for 5 min (Fig. 1a). All VMR experiments were conducted on 7 dpf between 9 am and 6 pm to minimize the effect of circadian rhythm on vision^82^.

### Light stimulus intensity

Light intensity of the ZebraBox LED spectrum was measured with a SpectriLight ILT950 Spectroradiometer (International Light Technologies). The total irradiance of the LED stimulus over the entire visible spectrum at 5% power output was 3.2 µW cm^−2^ (0.0063 µW cm^−2^ at 500 nm wavelength). The corresponding illuminance was 4.5 lx. The light intensity was further reduced by fitting neutral-density filters in the light path as described above. These neutral-density filters did not alter the color spectrum of the LED light (Supplementary Fig. S1). The light intensity with neutral-density filters was calculated by multiplying the light intensity emitted by the machine with the transmittance of each neutral-density filter. The irradiance of the final scotopic stimulus used in this study was 0.005 µW cm^−2^ (1.80e−5 µW cm^−2^ at 500 nm). The corresponding illuminance was 0.01 lx.

### Y79 cell culture and cAMP assays

The human Y79 cell line was obtained from American Type Culture Collection (ATCC HTB-18). These cells were cultured in RPMI-1640 Medium (ATCC, 30-2001) with 15% Fetal Bovine Serum (ATCC, 30-2020) at 37 °C with 5% CO_2_. To measure cAMP levels in the cells, the GloSensor Technology − 22F cAMP plasmid (Promega, E2301) was used with GloSensor Assay Reagent (Promega, E1290). Four million cells were seeded into 10-cm dishes with 10 mL of Opti-MEM Reduced Serum Medium (ThermoFisher, 31,985,062) for transfection. The cells were transfected with 20 µg of GloSensor plasmid utilizing X-tremeGENE HP DNA Transfection Reagent (MilliporeSigma, 6,366,244,001) at a 2:1 ratio of plasmid to X-tremeGENE reagent, according to manufacturer’s instructions. These cells were transfected for 24 h, and then they were transferred back into RPMI for another 24 h. Twenty-five thousand cells were then seeded into a low-volume white 384-well plate per well (Greiner Bio-one, 784,080). The cAMP assay was carried out according to the GloSensor protocol for suspension cells. Cells were either treated with DMSO or carvedilol for 20 min at room temperature prior to treatment with isoproterenol. Luminosity was recorded 20 min after drug or DMSO vehicle addition with a FlexStation 3 Multi-Mode Microplate Reader (Molecular Devices).

### Data visualization and statistical analysis

#### General data and statistics

All standard statistical analyses were performed with R version 3.6.0^83^.

#### VMR data

Raw data from the VMR assay was processed and extracted by Data Workshop (ViewPoint Life Sciences). Data figures were created using *ggplot2* package in R^84^. The VMR data were normalized for baseline activity, light intensity variation per well, and batch effect (i.e. biological replicate) by linear-regression models as previously described^56^. Additionally, offset values were added to the normalized activity to prevent negative values in displacement.

To determine if each VMR replicate from drug-treated Q344X larvae during drug screening was consistent with the other replicate, a high-dimensional nonparametric multivariate test^57^ was performed. This test was chosen because the number of observations (i.e. sample size) for each VMR is less than the dimension of the dataset. The dimension is the length of the time period used in the analysis and the sample size is the number of drug-treated larvae. The High-Dimensional Hypothesis test was implemented in the R package *HDtest*.

The Hotelling’s T-squared test^85^ was used to test significant changes in zebrafish displacement from 1 to 30 s after the light change. This test is the multivariate version of the T-test which follows the F-distribution. The test statistic for the Hotelling’s T-squared test is calculated as: ${\text{F}} = \frac{{{\text{n}}_{1} + {\text{n}}_{2} - {\uprho } - 1}}{{{\text{p}}\left( {{\text{n}}_{1} + {\text{n}}_{2} - 2} \right)}}{\text{T}}^{2} \sim {\text{F}}_{{{\text{p}},{\text{n}}_{1} + {\text{n}}_{2} - {\text{p}} - 1}}$ where $n_{1}$ and $n_{2}$ are the sample size. ρ is the dimension which is the time interval used in the analysis. The Hotelling’s T-squared test was used for VMR analysis due to a number of advantages: 1. The Type I error rate is controlled. 2. The relationship between multiple variables is considered. 3. It can generate an overall conclusion even if multiple (single) t-tests are inconsistent. The null hypothesis for the experiment is the group means for all response variables are equal which means the mean vector of the distance travelled for the two chosen groups are the same $\left( {\mu_{1} = \mu_{2} } \right)$. The Hotelling T-squared test analysis was performed on the R package *Hotelling* with some reshape of the dataset.

#### Y79 cAMP data

Luminosity data obtained from the Y79 cell line was analyzed and plotted using Graphpad Prism (version 8, GraphPad Software). Data were plotted with the non-linear fit method under “log(agonist) vs. response—Variable slope (three parameters)”. pEC50 (negative log of half maximal effective concentration) and pIC50 (negative log of half maximal inhibitory concentration) were calculated through the above-mentioned non-linear fit.

## Supplementary Information

Supplementary Information

## Data Availability

The raw zebrafish behavioral data is available on the Harvard Dataverse https://doi.org/10.7910/DVN/JYLWH1. The R scripts to reproduce the analyses and plots reported in this paper are available in GitHub https://github.com/zhanzmr/Zebrafish_Model.
