# Supplementary material for: Drug screening with zebrafish visual behavior identifies carvedilol as a potential treatment for an autosomal dominant form of retinitis pigmentosa
Source: Sci Rep. 2021 Jun 1;11:11432. doi: 10.1038/s41598-021-89482-z (PMC8169685; doi:10.1038/s41598-021-89482-z)

**Drug Screening with Zebrafish Visual Behavior Identifies Carvedilol as a Potential Treatment for an Autosomal Dominant form of Retinitis Pigmentosa**

Logan Ganzen^1,2^; Mee Jung Ko^2,3^; Mengrui Zhang^4^; Rui Xie^5^; Yongkai Chen^4^; Liyun Zhang^6^; Rebecca James^1^; Jeff Mumm^6^; Richard van Rijn^2,3,12,13^; Wenxuan Zhong^4^; Chi Pui Pang^7^; Mingzhi Zhang^8*^; Motokazu Tsujikawa^9,10^*; Yuk Fai Leung^1,11,12,13^*

1) Department of Biological Sciences, Purdue University, West Lafayette, IN, 47907

2) Purdue University Life Sciences Program, Purdue University, West Lafayette, IN, 47907

3) Department of Medicinal Chemistry and Molecular Pharmacology, Purdue University, West Lafayette, IN, 47907

4) Department of Statistics, University of Georgia, Athens, GA, 30602

5) Department of Statistics and Data Science, University of Central Florida, Orlando, FL, 32816

6) Wilmer Eye Institute, John Hopkins School of Medicine, Baltimore, MD, 21205

7) Department of Ophthalmology and Visual Sciences, Chinese University of Hong Kong, Hong Kong.

8) Joint Shantou International Eye Center, Shantou University and the Chinese University of Hong Kong, Shantou, China

9) Department of Ophthalmology, Osaka University Graduate School of Medicine, Osaka, Japan

10) Department of Clinical Laboratory and Biomedical Sciences, Osaka University Graduate School of Medicine, Osaka, Japan

11) Department of Biochemistry and Molecular Biology, Indiana University School of Medicine Lafayette, 625 Harrison Street, West Lafayette, IN 47907

12) Purdue Institute for Integrative Neuroscience, 610 Purdue Mall, Purdue University, West Lafayette, IN 47907

13) Purdue Institute for Drug Discovery, 610 Purdue Mall, Purdue University, West Lafayette, IN 47907

*Asterisk indicates corresponding authors.

Corresponding Author Emails:

Yuk Fai Leung: yfleung@purdue.edu

Motokazu Tsujikawa: moto@ophthal.med.osaka-u.ac.jp

Mingzhi Zhang: zmz@jsiec.org

**Supplemental Figure 1:**

Spectrum of the LED light source used to evoke VMR in the zebrafish larvae. (a) The light spectrum of the LED at 100% power output891 lux (red trace). A single neutral-density (ND) filter reduces the light outputapproximately by 60% (40% transmittance) to 365 lux without altering the spectrum (black trace). (b) Spectrum of the LED light source at 5% power is 4.5 lux. In this study, 7 ND filters were stacked upon the light source at 5% power output to create a scotopic light source that was approximately 0.01 lux.

**Supplemental Figure 2:**

(a) Both WT and Q344X larvae displayed a photopic light-on VMR. The response of WT (black trace) and Q344X (red trace) larvae at 891 lux. The light was turned on at Time = 0. Each trace shows the average larval displacement of 3 technical replicates with 32 larvae per replicate. The corresponding color ribbon indicates ± 1 s.e.m. (b) Both WT and Q344X larvae display a photopic light-off VMR. The photopic light-off VMR of WT (black trace) and Q344X (red trace) larvae at 891 lux. The light was turned off at Time = 0. Each trace shows the average larval displacement of 3 technical replicates with 48 larvae per replicate. The corresponding color ribbon indicates ± 1 s.e.m. (c) Both WT and Q344X larvae did not display a scotopic light-on VMR. The response of WT (black trace) and Q344X (red trace) larvae at 0.01 lux. The light was turned on at Time = 0. Each trace shows the average larval displacement of 18 biological replicates with 48 larvae per condition per replicate. The corresponding color ribbon indicates ± 1 s.e.m. (d) Boxplot of the average larval displacement of WT and Q344X larvae one second after scotopic light onset. The average displacement of WT larvae (*µ* ± s.e.m. *)*: 0.129 ± 0.013 cm, N = 18) was not significantly different from Q344X larvae (0.130 ± 0.013 cm, N = 18) (Welch’s Two Sample t-test, T = 0.08, df = 33.8, p-value = 0.93).

**Supplemental Figure 3:**

(a) *rho*:*NTR* larvae displayed a photopic light-on VMR with and without MTZ treatment. The response of *rho:NTR* larvae (black trace) and *rho:NTR* with MTZ treatment (red trace) at 891 lux. Light onset occurred at T =0. Each trace represents the average displacement of 4 biological replicates of N = 24 larvae. The corresponding color ribbon indicates ± 1 s.e.m. (b) *rho:NTR* larvae displayed a photopic light-off VMR. The light-off VMR of *rho:NTR* larvae (black trace) and *rho:NTR* with MTZ treatment (red trace) at 891 lux. Light onset occurred at T =0. Each trace represents the average displacement of 4 biological replicates of N = 24 larvae. The corresponding color ribbon indicates ± 1 s.e.m. (c) The *rho:NTR* larvae did not display a strong scotopic light-on VMR or without MTZ treatment. The scotopic light-on VMR of *rho:NTR* larvae (black trace) and *rho:NTR* with MTZ treatment (red trace) at 0.01 lux. Light onset occurred at T =0. Each trace represents the average displacement of 4 biological replicates of N = 24 larvae. The corresponding color ribbon indicates ± 1 s.e.m.

**Supplemental Figure 4:**

(a) Photopic light-on VMR of Q344X larvae treated with DMSO at 3 dpf (red trace) or carvedilol at 3 dpf (blue trace). Carvedilol does not have a significant effect on the photopic light-on VMR (Hotellings T-squared test, N = 3 replicates of 24 larvae, T = 44.1, df = 30, p-value = 0.273). (b) Photopic light-off VMR of Q344X larvae treated with DMSO at 3 dpf (red trace) or carvedilol at 3 dpf (blue trace). Carvedilol does not have a significant effect on the photopic light-off VMR (Hotellings T-squared test, N = 3 replicates of 24 larvae, T = 30.4, df = 30, p-value = 0.745).


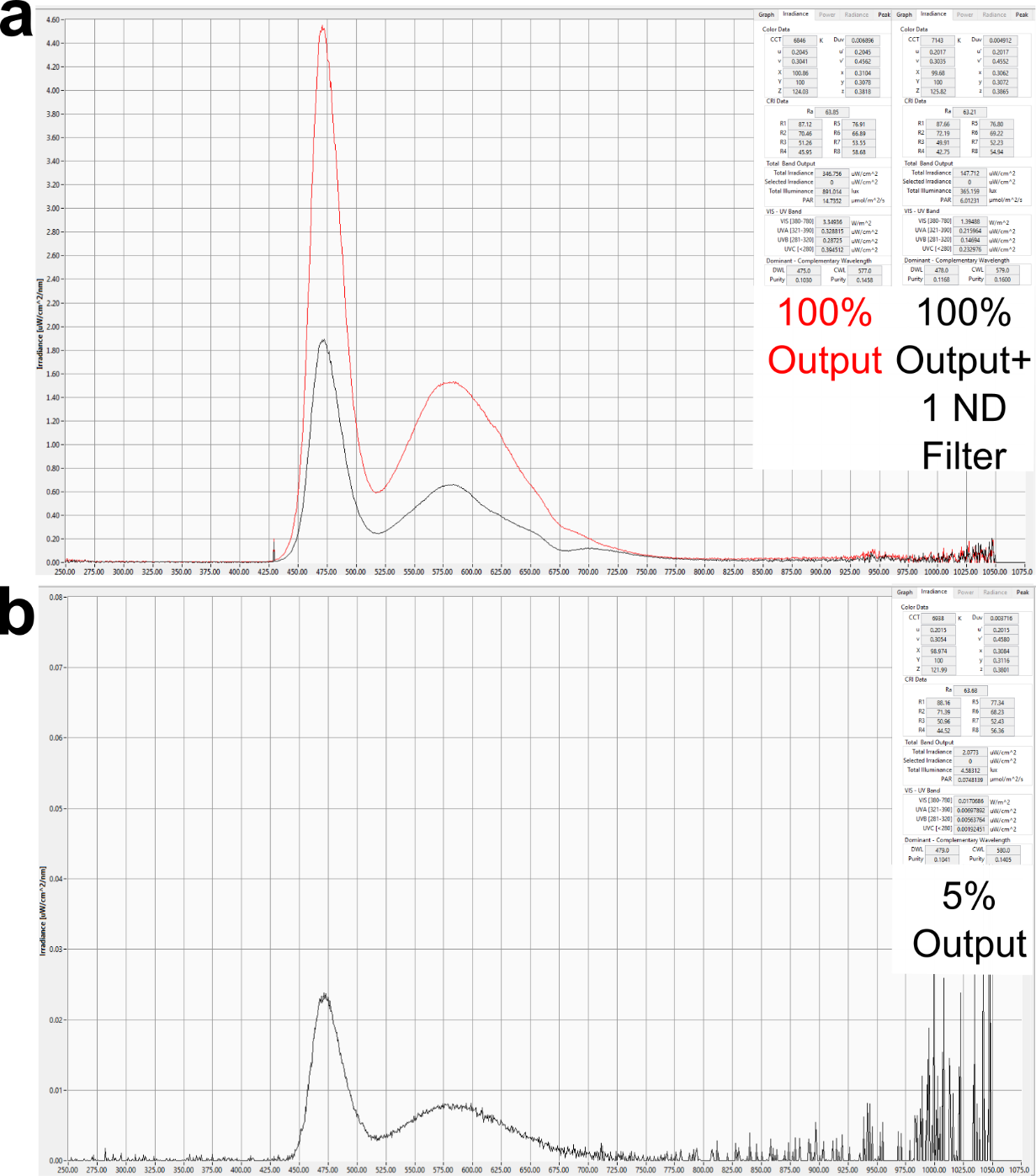
Supplemental Figure 1


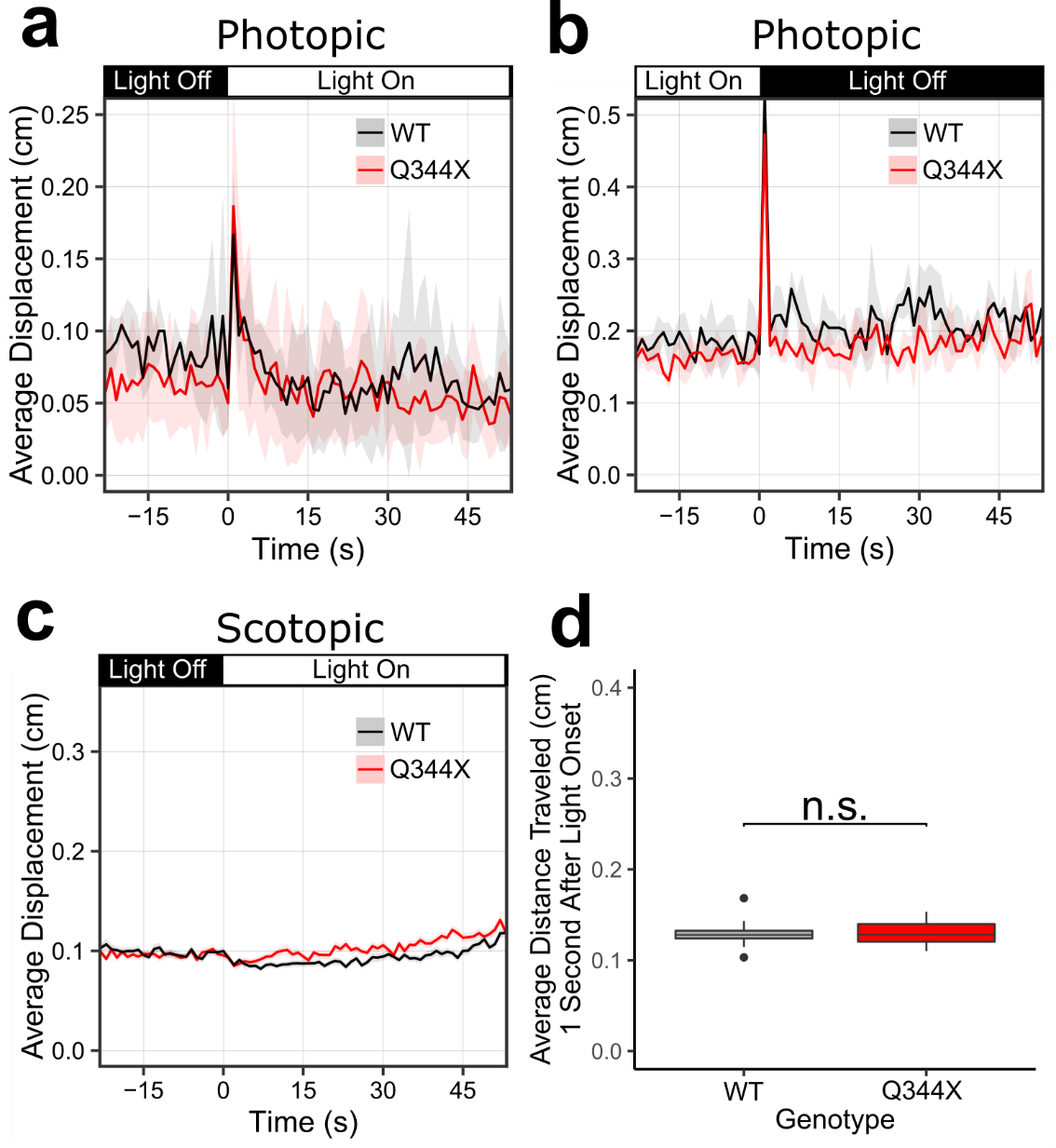
Supplemental Figure 2


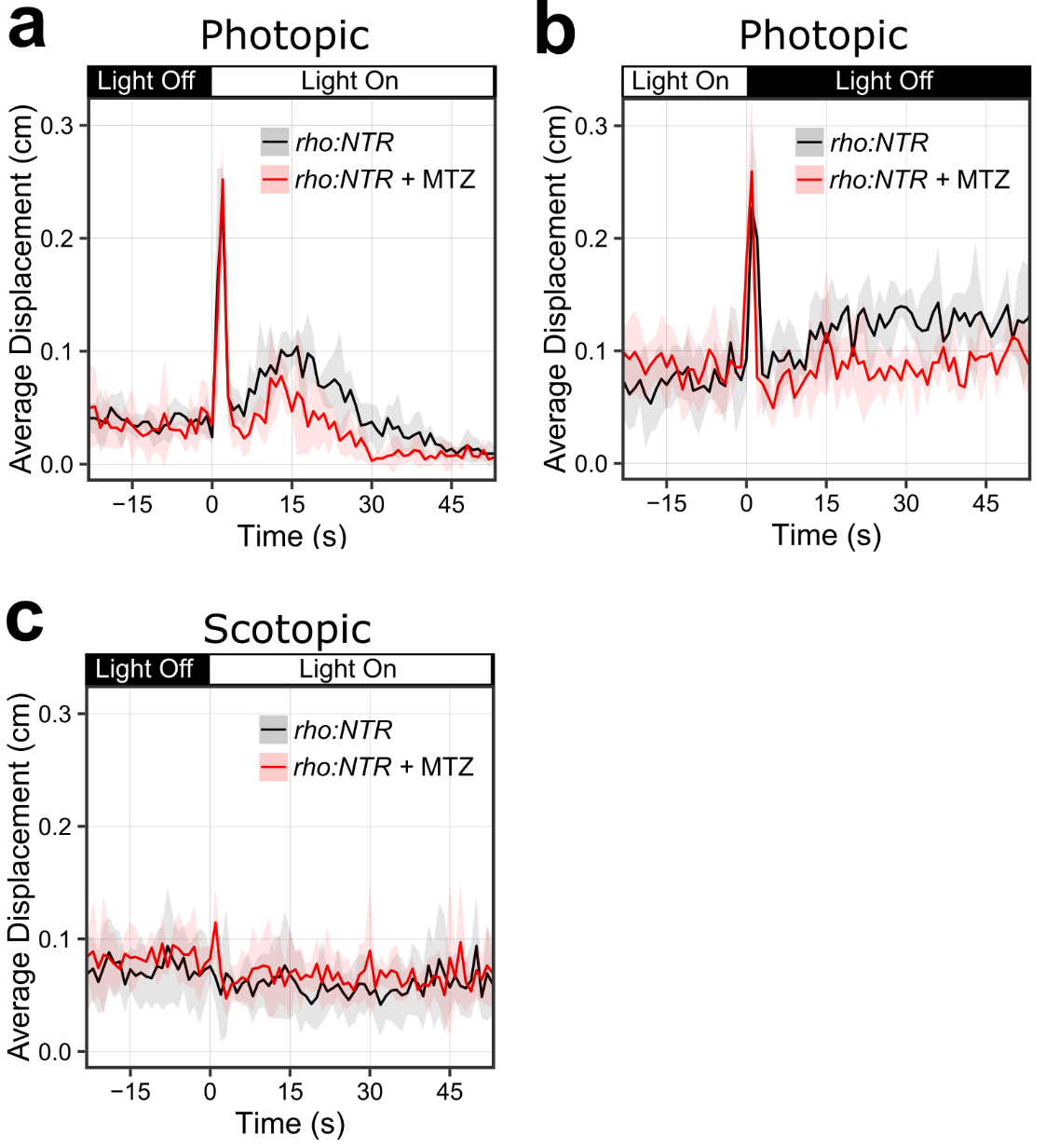
Supplemental Figure 3

Supplemental Figure 4


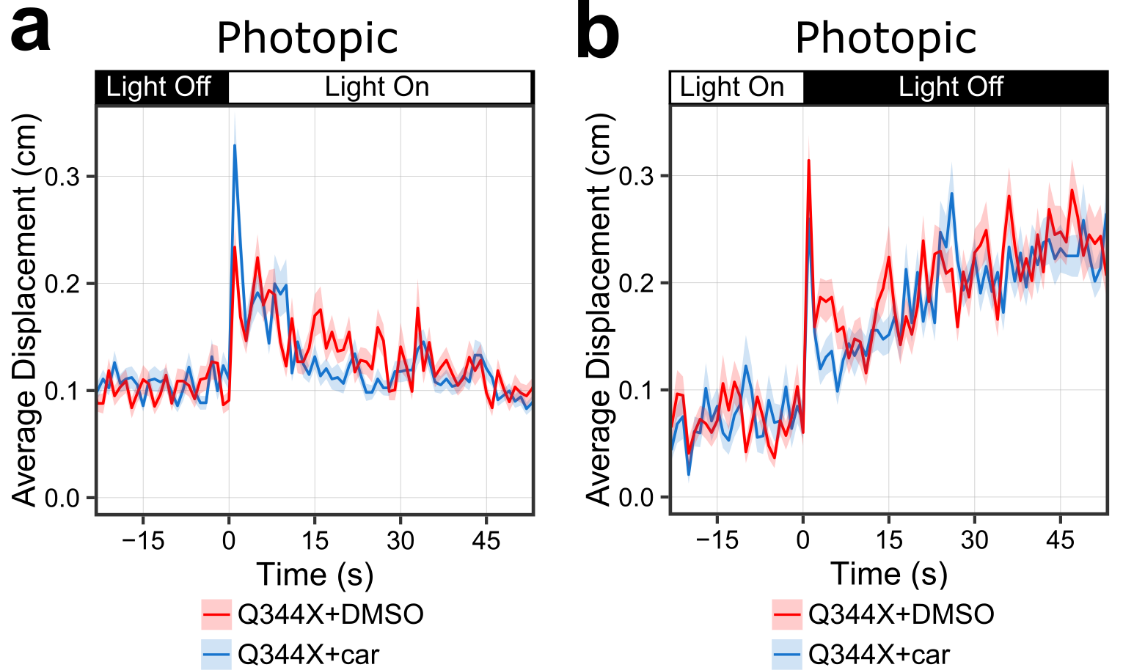

Supplement: Supplementary file 1 — Supplementary Information [file 41598_2021_89482_MOESM1_ESM.docx]
